# Enhancement of the edible quality and shelf life of soft‐boiled chicken using MAP

**DOI:** 10.1002/fsn3.1447

**Published:** 2020-02-18

**Authors:** Shaolin Deng, Ming Li, Huhu Wang, Xinglian Xu, Guanghong Zhou

**Affiliations:** ^1^ Jiangsu Collaborative Innovation Center of Meat Production and Processing Quality and Safety Control College of Food Science and Technology Nanjing Agricultural University Nanjing China

**Keywords:** modified atmosphere packaging, sensory evaluation, shelf life, soft‐boiled chicken

## Abstract

In order to commercialize soft‐boiled chicken, a traditional Asian food, this work was performed to evaluate the effect of the packages on the edible quality. Soft‐boiled chickens were packaged with air packaging (AP) or modified atmosphere packaging (MAP, MN:100% N_2_ and MC:30%CO_2_/70%N_2_) conditions. Total viable counts reached the acceptable limit of 4.90 Log CFU/g at 1.5, 2.5, and 4 days of storage under AP, MN, and MC, respectively. However, sensory acceptance limits, based on overall acceptance by the professional panelists, were not reached until one day later. The TVB‐N content of MAP was lower than that of the AP. The finding indicated that the shelf life of soft‐boiled chicken could be extended by two days using MAP with CO_2_. These findings will provide basic reference for the packaging of cooked meat and also provide information on poultry processing techniques that could be applied at commercial scales.

## Graphical abstract

According to our microbiology determination and sensory evaluation results, MAP treatment with 30% CO_2_/70% N_2_ can prolong the shelf life of SC to 4 days.

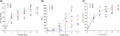

## 1 INTRODUCTION

The Yellow‐feathered broiler, which has a distinctive flavor and texture profiles compared to other commercial broilers such as Ross, Arbor Acres, and Cobb Broiler, is widely considered the primary raw meat in the manufacturing of soft‐boiled chicken (SC; Jayasena et al., 2015). The soft‐boiled chicken, also named White‐Cut Chicken, is a popular traditional poultry product in Asian‐Pacific areas, with a similar reputation as Beijing roasted duck. SC is traditionally characterized by white flesh with a slight oily‐yellow appearance, and the addition of dipping sauce can usually improve the unique flavor. Only boiling and cold‐dipping are required to manufacture SC, which is not challenging to exercise both at industrial scales and prepared at kitchen. Beside 5% of sodium chloride, which is indispensable to the manufacture of SC, there is no other ingredient. Additionally, customers preferred to purchase the ready‐to‐eat SC that requires minimal processing at home.

The original flavor and tender profiles of raw chicken contribute greatly to the widespread acceptance of SC (Chen, Guo, Xu, Lu, & Zhang, 2014). However, poultry products are generally sterilized at 121°C for 10 min or more subsequent to packaging (Sun et al., 2016), which could greatly reduce the special flavor and texture of SC. The short shelf life of SC without sterilization, usually less than one day at ambient temperature, has become an obstacle for industrialization of SC. Modified atmosphere packaging (MAP) has been widely demonstrated to extend the shelf life of meat products (Azlin‐Hasim, Cruz‐Romero, Morris, Cummins, & Kerry, 2015; Latou, Mexis, Badeka, Kontakos, & Kontominas, 2014; Limbo et al., 2013). Several meat companies in China, including Zhou Heiya International Holdings Limited and Shandong Dezhou Braised Chicken Company Limited, have adapted MAP to extend the shelf life of poultry products. Using MAP with CO_2_ could obviously inhibit microbial growth (Zhang, Wang, Li, Li, & Xu, 2015), while the primary function of N_2_ in MAP is to maintain packaging integrity.

The objectives of the present study were to evaluate the shelf life of SC under air packaging (AP) and two different MAP conditions (modification with 100% N_2_ [MN], and modification with 30% CO_2_/70% N_2_ [MC; Zhang et al., 2015]). The finding could help industrialize an improved packaging and processing technique for poultry products.

## 2 MATERIALS AND METHODS

### 2.1 Sample preparation

Sixty‐four yellow‐feathered raw chickens (115 days of age) were randomly selected from a local slaughterhouse (Lihua Food Co. Ltd.). Chicken carcasses weighed about 1.0 kg were transported to the laboratory at 4 ± 1°C within one hour and were processed immediately.

To prepare the soft‐boiled chickens, carcasses were submerged in boiling water with 5% sodium chloride for 2 s (three times) and then immersed in ice water for 10 s. Carcasses were then resubmerged in 95°C water for 20 min and then cold‐dipped (placed into ice water for chilling) for 15 min. The prepared soft‐boiled chickens were randomly assigned to three packaging treatments (AP, MN, and MC), and the headspace in each packaged boxes was appropriate 45%. AP samples were packaged in a polyethylene film with an oxygen permeability of 14,483 cm^3^/(m^2^·day·atm), a CO_2_ permeability of 63,683 cm^3^/(m^2^·day·atm), and a water vapor permeability of 54 g/(m^2^·day·atm). MN and MC samples were packaged in low‐density polyethylene/polyamide/low‐density polyethylene (LDPE/PA/LDPE) barrier pouches (1 fillet per pouch), with a thickness of 25 mm and an oxygen permeability of 24 cm^3^/(m^2^·day·atm) at 0% relative humidity and 23°C, CO_2_ permeability of 78 cm^3^/(m^2^·day·atm) at 0% RH and 23°C, and water vapor permeability of 44 g/(m^2^·day) at 100% RH and 38°C. All samples were stored at 12 ± 0.5°C (Compressor‐Cooled Incubator ICP 260), and sampled at 0, 1, 2, 3, 4, and 5 days. AP samples were not analyzed on the fifth day because their shelf lives had been surpassed.

### 2.2 Sensory evaluation

Sensory evaluation was performed by a professional panel (Al‐Juhaimi et al., 2019; Chen et al., 2019). A total of 10 panelists, who were experienced in the sensory analysis of meat products, were trained following the Chinese standard GB/T 22210‐2008 (Criterion for sensory evaluation of meat and meat products) to learn how to recognize and quantitate the profiles of structure, appearance, odor, texture, surface slime, and overall acceptability. First, they were trained on several kinds of sensory profiles of soft‐boiled chickens to evaluate the threshold of acceptance. Then, triangle test was used to determine the sensitivity of the panels of each profile. Finally, they were trained using the fresh soft‐boiled chickens and asked to memorize its profiles, particular of odor and structure. The training was not performed until the data from the panelists kept at a stabilized level.

The soft‐boiled chickens were sliced into pieces with thick of approximately 10 mm and placed on a white ceramic plate. Each treatment was identified with a three‐digit random code. Panelists were instructed to rinse their mouths with warm water between each sample to reduce cross‐influence. Each panelist was asked to evaluate the sensory attributes of the SC including structure, appearance, odor, texture or surface slime, and overall acceptability. A 9‐point hedonic scale was used to score these attributes (Zhang et al., 2015): excellent, 9; very good, 8; good, 7; acceptable, 6; between acceptable and unacceptable, 5; slightly unacceptable, 4; moderately unacceptable, 3; very much unacceptable, 2; and extremely unacceptable, 1, a score of 6 was used as the limit of acceptability.

### 2.3 Headspace gas analysis

The composition of the headspace gas in each packaging box was measured according to the method described by Al‐Nehlawi, Saldo, Vega, and Guri (2013) using an oxygen and carbon dioxide analyzer (MAPY 4.0, Witt‐Gasetechnik GmbH & Co KG).

### 2.4 pH, color, and TVB‐N measurements

The pH values were determined according to the method proposed by Wang et al. (2017). The skin color of soft‐boiled broiler was measured with the previous procedures using a chromameter (Minolta CR400; Wang, Qin, Li, Xu, & Zhou, 2019). A white color standard reference was used for instrument calibration. The average values of light (*L* ^*^), red (*a* ^*^) and yellow (*b* ^*^) of skin from chest were selected to access the color of the samples. Total volatile basic nitrogen (TVB‐N) was determined according to the China National Standard—Method for the analysis of hygienic standard of meat and meat products (GB/T 5009.44‐2003). The TVB‐N contents were expressed as mg per 100 g.

### 2.5 Microbiological analysis

Immediately after aseptically opening the packages, each 25 g of sample was weighted and placed in stomacher bags containing 225 ml of saline (0.85%). Total viable counts (TVCs) and total coliforms were determined according to the China National Food Safety Standard methods (GB 47892‐2010/GB 47893‐2010). TVCs were determined using a plate count agar (PAC, Land Bridge) after incubation for 2 days at 37°C. Coliforms were determined using lauryl sulfate tryptose broth (LST, Hope Bio) and brilliant green lactose bile broth (BLGB, Hope Bio) incubation for 2 days at 37°C. Lactic acid bacteria (LAB) were determined according to the method proposed by Chouliara, Karatapanis, Savvaidis, and Kontominas (2007). LAB was counted using MRS agar (Oxoid code CM 0361) after incubation for 3 days at 25°C.

### 2.6 Statistical analysis

Each indicator was repeated four times. TVC and LAB data were transformed Log 10 (CFU/g). The number of total coliforms was expressed as the most‐probable number (MPN). Data were analyzed with one‐way ANOVAs and Duncan's multiple range tests. *p* Values <.05 were considered statistically significant.

## 3 RESULTS AND DISCUSSION

### 3.1 Sensory evaluation

Meat structure, appearance, odor, and surface slime are critical sensory attributes that affect consumer judgment when purchasing SC products. These sensorial characteristics offer a glimpse of the prospective shelf life of SC products. Sensory scores for the acceptance gradually decreased with storage time in this work (Table 1). The samples with MC obtained the highest scores for all attributes compared to the other treatments, and the differences were significantly different on the and after the fourth day. The overall acceptance of the MC samples did not go below the limit of acceptability (6 points), whereas the odor acceptance was close to the accepted limit. The AP, MN, and MC treatments reached the limit of acceptability for odor on days 2–3, 3–4, and 5, respectively. For the specific indicators of sensory evaluation, the general trends were similar to those of overall acceptability.

**Table 1:** Changes in the sensory properties of soft‐boiled chicken with different packaging

Sensory	Storage time (days)					
	0	1	2	3	4	5
Structure						
AP	9.00 ± 0.00^a^	7.50 ± 0.47^Bb^	7.55 ± 0.44^Ab^	5.95 ± 0.72^Cc^	4.30 ± 0.82^Cd^	—
MN	9.00 ± 0.00^a^	8.00 ± 0.57^Ab^	7.50 ± 0.47^Ac^	6.55 ± 0.37^Bd^	5.50 ± 0.71^Be^	5.50 ± 0.62^Be^
MC	9.00 ± 0.00^a^	8.00 ± 0.41^Ab^	7.60 ± 0.52^Ab^	7.05 ± 0.16^Ac^	6.60 ± 0.91^Acd^	6.50 ± 0.71^Ad^
Appearance						
AP	9.00 ± 0.00^a^	7.45 ± 0.69^Ab^	6.80 ± 0.75^Bbc^	6.30 ± 1.09^Bc^	4.70 ± 0.67^Cd^	—
MN	9.00 ± 0.00^a^	7.45 ± 0.90^Ab^	7.45 ± 0.64^Ab^	6.80 ± 0.59^ABb^	5.50 ± 0.53^Bc^	5.45 ± 1.12^Bc^
MC	9.00 ± 0.00^a^	7.90 ± 0.74^Ab^	7.50 ± 1.08^Abc^	7.00 ± 0.47^Acd^	6.40 ± 0.70^Ade^	6.20 ± 0.63^Ae^
Odor						
AP	9.00 ± 0.00^a^	6.60 ± 0.53^Bb^	6.50 ± 0.67^Bb^	4.80 ± 0.75^Bc^	3.65 ± 0.58^Cd^	—
MN	9.00 ± 0.00^a^	7.35 ± 0.97^Ab^	6.85 ± 0.58^Bb^	6.15 ± 0.58^Ac^	5.00 ± 0.41^Bd^	4.90 ± 0.88^Bd^
MC	9.00 ± 0.00^a^	7.40 ± 0.94^Ab^	7.30 ± 0.71^Ab^	6.55 ± 0.69^Ac^	6.50 ± 0.67^Ac^	6.05 ± 0.60^Ac^
Surface slime						
AP	9.00 ± 0.00^a^	7.40 ± 0.32^Bb^	6.65 ± 0.41^Bc^	5.90 ± 0.81^Bd^	4.60 ± 0.52^Ce^	—
MN	9.00 ± 0.00^a^	7.65 ± 0.41^Ab^	6.95 ± 0.37^Bc^	6.50 ± 0.33^Ac^	5.50 ± 0.71^Bd^	5.00 ± 0.94^Be^
MC	9.00 ± 0.00^a^	7.65 ± 0.37^Ab^	7.45 ± 0.37^Ab^	6.70 ± 0.42^Ac^	6.35 ± 0.97^Ac^	6.45 ± 0.64^Ac^
Overall acceptance						
AP	9.00 ± 0.00^a^	7.15 ± 0.63^Bb^	6.80 ± 0.54^Cb^	5.55 ± 0.55^Cc^	4.25 ± 0.82^Cd^	—
MN	9.00 ± 0.00^a^	7.65 ± 0.58^Ab^	7.20 ± 0.26^Bb^	6.50 ± 0.47^Bc^	5.60 ± 0.52^Bd^	5.25 ± 0.95^Bd^
MC	9.00 ± 0.00^a^	8.00 ± 0.58^Ab^	7.60 ± 0.66^Abc^	7.15 ± 0.47^Ac^	6.55 ± 0.72^Ad^	6.45 ± 0.50^Ad^

Note

Values are expressed as the means ± standard deviations (*n* = 4). Different upper‐case letters (across rows) and different lower‐case letters (within sensory property) indicate significant differences (*p* < .05). AP, air packaging; MN, modified atmosphere packaging with 100% N_2_, and MC, modified atmosphere packaging with 30% CO_2_/70% N_2_.

Microbial activities are usually responsible for unacceptable odors and surface slime (Singh, Wani, Saengerlaub, & Langowski, 2011). However, the shelf life based on TVC was one day shorter than the shelf life estimated using sensorial attributes. Furthermore, the results of sensory evaluation were not entirely in agreement with microbiological data. Given that specific spoilage microorganisms are responsible for inducing spoilage, rather than the values indicated by TVC, the sensorial attributes in this work are acceptable (Rossaint, Klausmann, Herbert, & Kreyenschmidt, 2014). Sensory evaluation performed by panelists could reflect the alterations of physical and chemical properties, but it is typically less sensitive and accurate than microbial evaluation.

### 3.2 Headspace gas analyses

**Figure 1:**
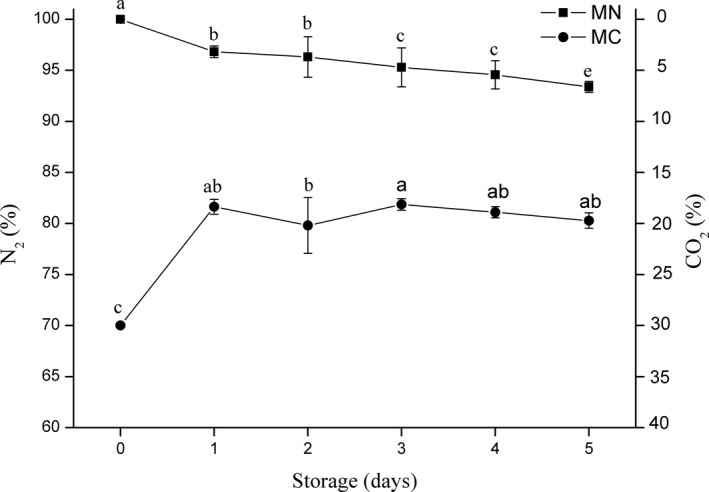
Headspace N_2_ and CO_2_ percentages of soft‐boiled chicken overtime in different package (MN = modified atmosphere packaging with 100% N_2_, and MC = modified atmosphere packaging with 30% CO_2_/70% N_2_). Error bars represent standard deviations of the mean (*n* = 4), and different letters indicate significant differences in gas concentrations between days (*p* < .05)

The relative content of N_2_ consistently decreased in the MN treatment (Figure 1), probably due to absorption into the meat (Al‐Nehlawi et al., 2013). Furthermore, anaerobic microorganisms can induce the decomposition of carbohydrates in tissue, producing CO_2_ and accounting for the decreased N_2_ content. Nevertheless, the decreasing of N_2_ after two days of storage was close to keep stable, while the proportion of CO_2_ continued to increase daily. This indicates that microbial activity may be vigorous and accelerated the spoilage of the SC.

In the MC packaging group, the rate of decrease in CO_2_ levels in the headspace was greater during the first 24 hr of storage, when the relative content reduced from 29.98% to 18.35% (Figure 1). This may be explained by the solubility of CO_2_ in the meat tissues of the SC (Al‐Nehlawi et al., 2013), although the collapsion of packaging was not determined in this work. Subsequently, the relative content of CO_2_ in the MC packaging treatment tended to be constant, implying that an equilibrium between the CO_2_ dissolving and the CO_2_ present in the package headspace was attained on one‐day storage. This result is consistent with the results of Mendes, Pestana, & Gonçalves, 2008). In addition, Rotabakk, Birkeland, Jeksrud, and Sivertsvik (2006) also reported the similar finding the proportion of CO_2_ dissolved in meat should decrease accordingly throughout storage periods. The parameters of MAP used in this study could cause little shrinkage and collapse of the packing film, which is usually induced by gas dissolution in MAP system with high proportion of CO_2_.

### 3.3 pH and color

An increase of pH values was observed overtime (Table 2). The AP samples had the fastest rates of increasing and were significantly higher than those of other treatments on the fourth day of storage. During the first four days of storage, the pH values of MC samples decreased slightly. This could be due to the high concentrations of CO_2_; the dissolution of CO_2_ in the first 24 hr after packaging (Al‐Nehlawi et al., 2013) was observed in the gas composition (Figure 1).

**Table 2:** Changes in pH and color values of soft‐boiled chicken with different packaging

Packaging	Storage times (days)					
	0	1	2	3	4	5
AP						
pH	6.25 ± 0.02^b^	6.21 ± 0.06^Bb^	6.32 ± 0.15^Ab^	6.31 ± 0.15^Ab^	6.89 ± 0.15^Aa^	—
*L* ^*^	74.37 ± 1.08^a^	71.24 ± 1.80^b^	68.62 ± 1.99^bc^	69.57 ± 0.80^bc^	67.20 ± 2.74^c^	—
*a* ^*^	0.32 ± 1.23	0.78 ± 0.41	1.18 ± 0.45	0.85 ± 0.69	2.03 ± 0.38	—
*b* ^*^	29.34 ± 2.60	25.76 ± 2.21	26.16 ± 1.81	24.28 ± 1.19	21.76 ± 3.61	—
MN						
pH	6.25 ± 0.02^bc^	6.32 ± 0.05^Ab^	6.14 ± 0.07^Bbc^	6.32 ± 0.11^Ab^	6.19 ± 0.05^Bc^	6.54 ± 0.33^Aa^
*L* ^*^	74.38 ± 1.08^a^	68.17 ± 1.46^b^	68.02 ± 2.09^b^	68.25 ± 1.76^b^	67.38 ± 2.09^b^	66.22 ± 1.26^b^
*a* ^*^	0.32 ± 1.23	2.23 ± 0.79	2.70 ± 0.89	1.85 ± 0.54	1.78 ± 0.67	2.43 ± 0.53
*b* ^*^	29.34 ± 2.60	26.85 ± 5.13	27.49 ± 1.27	27.08 ± 1.69	24.67 ± 5.24	23.15 ± 0.87
MC						
pH	6.25 ± 0.02^ab^	6.23 ± 0.05^Bab^	6.22 ± 0.02^ABab^	6.16 ± 0.04^Ab^	6.15 ± 0.08^Bb^	6.29 ± 0.11^Ba^
*L* ^*^	74.38 ± 1.08^a^	66.77 ± 5.06^b^	67.48 ± 2.40^b^	68.41 ± 0.76^b^	68.73 ± 1.21^b^	67.01 ± 1.72^b^
*a* ^*^	0.32 ± 1.23	2.07 ± 0.77	1.60 ± 1.03	1.40 ± 0.66	2.68 ± 0.43	2.88 ± 0.55
*b* ^*^	29.34 ± 2.60	26.73 ± 1.35	25.30 ± 3.02	27.87 ± 3.03	25.75 ± 0.95	25.64 ± 0.89

Note

Values are expressed as means ± standard deviations (*n* = 4). AP, air packaging; MN, modified atmosphere packaging with 100% N_2_, and MC, modified atmosphere packaging with 30% CO_2_/70% N_2_.

When coming to the surface color, the *L* ^*^ values were significantly lower after one day of storage compared to initial values and decreased overtime (Table 2). However, there was no difference in *L* ^*^ values between tested groups. Neither treatment nor storage time could affect *a* ^*^ and *b* ^*^ values, although a fast decreasing trend was observed in AP group compared with other two groups. These results are partially agree with those reported by Rotabakk et al. (2006), who also found that there was no significant effect of high concentrations of CO_2_ on surface color of chicken breasts. Werner, Janisch, and Wicke (2008) also reported a similar variation in the lightness of poultry species during storage. Nevertheless, these color variations can seldom be recognized with the naked eye. It seems that increasing the dissolved CO_2_ content of SC meat or skin does not influence the final surface color.

### 3.4 TVB‐N values

**Figure 2:**
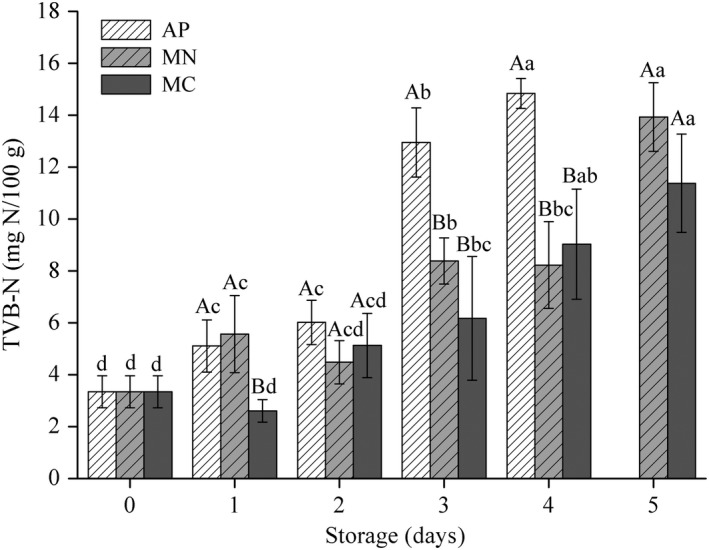
Total volatile basic nitrogen (TVB‐N) concentrations of soft‐boiled chicken overtime packaged using different methods (AP = air packaging, MN = modified atmosphere packaging with 100% N_2_, and MC = modified atmosphere packaging with 30% CO_2_/70% N_2_). Error bars represent standard deviations of the mean (*n* = 4). Different lower‐case letters indicate significant differences within packaging treatment, and different upper‐case letters indicate significant differences within a given day (*p* < .05)

TVB‐N is widely used as an indicator for meat spoilage, because protein and nonprotein nitrogenous compounds in meat, such as amino acids and nucleotide catabolites, can be degraded into amines and ammonia by microorganisms and endogenous enzymes (Liu, Liang, Xia, Regenstein, & Zhou, 2013). The standard value of TVB‐N for accepted meat is considered to be 15 mg/100 g in China (GB 16869‐2005). After 2 days of storage, the TVB‐N values of the AP samples increased rapidly and were significantly higher than the values of the other two treatments, increasing from an initial content of 3.34 mg/100 g–14.84 mg/100 g on the fourth day (Figure 2). The TVB‐N values of the MAP treatments were lower than the acceptance limit throughout the storage, with the MN and MC samples reaching 13.93 and 11.38 mg N/100 g, respectively. The TVB‐N trends in this study were less drastic than those of raw meat (Zhang, Wang, Li, Wu, & Xu, 2016).

The primary reason for the rapid increase of TVB‐N values during storage is contributed to the growth of microorganisms. Consequently, the capacity of protein catabolism would increase, along with an accelerated rate of product spoilage. However, the species of microorganisms found in the MAP samples were anaerobic bacteria, such as LAB, whose capacity to break down proteins is limited by the packaging condition. As a result, the TVB‐N values in MAP group were relatively low. The observed decrease in the TVB‐N values of the MC treatment during the first 1–2 days of storage might be ascribed to the buffering capacity of the meat matrix itself, which would dissolve and neutralize some of the basic nitrogen‐containing substances.

### 3.5 Microbiological analyses

**Figure 3:**
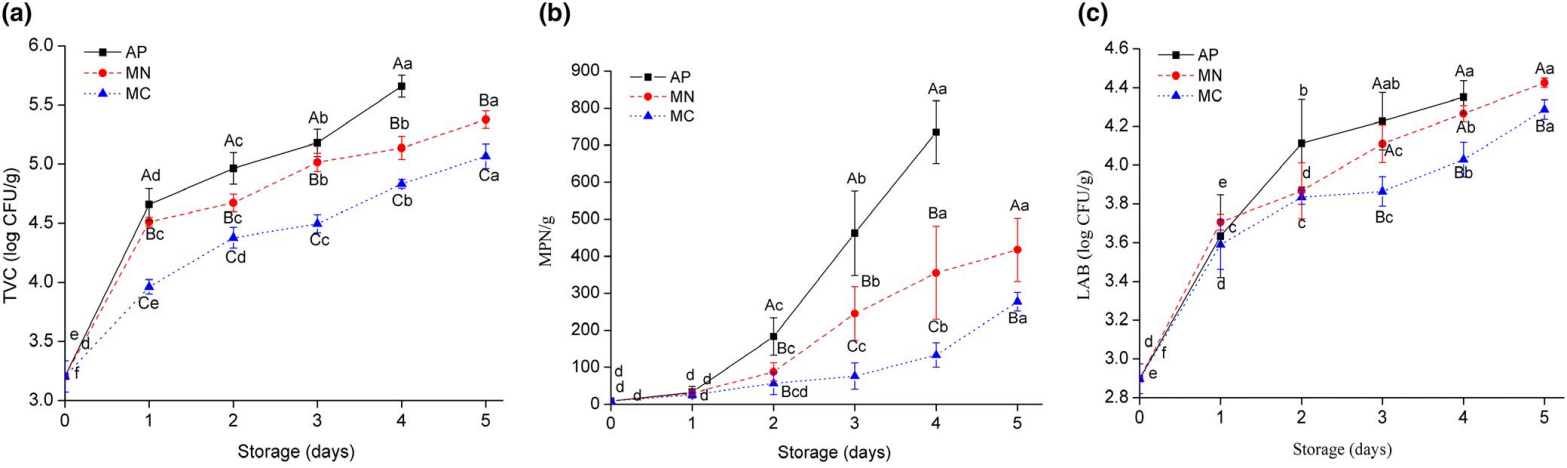
Changes in total viable counts (TVC) (a); coliforms (b); and lactic acid bacteria (LAB) (c) of soft‐boiled chicken with different packaging (stored at 12°C). Error bars represent standard deviations of the mean (*n* = 4). Different upper‐case letters indicate significant differences between treatments for a given storage time, and different lower‐case letters indicate significant differences overtime within different packaging treatments (*p* < .05)

A score of 4.90 log CFU/g is considered the upper threshold for pot‐stewed meat by the China National Food Safety Standard methods (GB2726‐2005). The TVC at day 0 was 3.20 log CFU/g, and the TVCs of AP, MN, and MC exceeded the accepted limit after 1–2, 2–3, and 4 days, respectively. The TVC values of MC were significantly lower than those of AP and MN samples (Figure 3a). The MC samples had a shelf life with almost 2.5 days longer than that of AP treatment.

A CO_2_ atmosphere can inhibit the growth of aerobic flora such as *Pseudomonas* (Ho, Huang, & McMillin, 2003; Rotabakk et al., 2006; Yang et al., 2016), which is the dominant microbe in poultry meat under air packaging conditions. In this study, the shelf lives of AP, MN, and MC were shorter than those of Patsias, Chouliara, Badeka, Savvaidis, and Kontominas (2006), who has studied the effects of air and MAP on chilled and precooked chicken products. The differences may be explained by the fact that the samples in this study were half‐chicken carcasses with skin, while other studies were excluded skin (Chouliara et al., 2007; Rokka, Eerola, Smolander, Alakomi, & Ahvenainen, 2004).

Coliforms are reliable indicators of the hygienic conditions of cooked meat products. The coliform threshold for pot‐stewed meat is 150 MPN/g according to the Food Safety Standard—Hygienic standard for cooked meat products (GB2726‐2005). The initial coliform populations of SC meat were 9.2 MPN/g. The AP samples exceeded the limit after 1–2 days of storage, while the MN and MC samples reached the threshold after 2–3 and 4–5 days of storage, respectively (Figure 3b). During days 0–1, no significant difference was observed in the MPN values of coliforms between each group. However, after the second day, coliform counts in the MC samples were significantly lower than those of the other groups. Most coliforms are aerobic and facultative anaerobic bacteria, so an anoxic atmosphere environment, especially one with CO_2_, is highly possible to reduce the growth rates of coliform bacteria compared to pure N_2_ conditions.

LAB usually represents the natural microflora of fresh chicken carcasses and products stored with AP or MAP (Meredith et al., 2014). The initial population of LAB was 2.89 log CFU/g, and these bacteria reproduced vigorously between 0 and 2 days in all groups. LAB includes facultative anaerobes that are more resistant to CO_2_ (Tsironi & Taoukis, 2010), suggesting that an oxygen‐deficient environment would have little impact on their growth. However, after three days of storage, the LAB values of MC samples were significantly lower than those of the AP and MN treatments. The finding indicates that 30% CO_2_ anaerobic atmosphere could reduce the growth rate of LAB, which is consistent with the results of Zhang et al. (2015), Zhang et al. (2016).

## 4 CONCLUSIONS

Basic on the observations of sensory evaluation, edible profile, and microbial loads, the finding demonstrated that MAP treatment with 30% CO_2_/70% N_2_ could prolong the shelf life of soft‐boiled chickens to four days during storage, as compared with air packaging and MAP with 100% N_2_. Although the scores of sensory evaluation were still within the acceptable limit, the total viable counts reached the limit of 4.90 log CFU/g at 4 days of under MAP with 30% CO_2_. In addition, the MAP with CO_2_ could greatly reduce the contents of TVB‐N. These findings will provide basic reference for the packaging of cooked meat and also provide information on the industrialization of traditional poultry foods.

## CONFLICT OF INTEREST

All authors declare that there is no conflict of interest.

## ETHICAL APPROVAL

There was no human or animal testing in this study.
